# Efficacy of *Nigella sativa* seeds oil in patients with Behcet’s disease: a double-blind randomized controlled trial 

**Published:** 2018

**Authors:** Hadiseh Kavandi, Mehrzad Hajialilo, Alireza Khabbazi

**Affiliations:** *Connective Tissue Diseases Research Center, Tabriz University of Medical Sciences, Tabriz, Iran*

**Keywords:** Behcet's disease, Nigella sativa, Iranian Behcet’s disease dynamic activity measure (IBDDAM), Behcet’s disease current activity form (BDCAF)

## Abstract

**Objective:**

*Nigella sativa* (NS) is a herbal medicine with anti-inflammatory and anti-oxidant functions. This study was designed to evaluate the effect of oral administration of NS seeds oil on the treatment of Behcet’s disease (BD).

**Materials and methods:**

In this double-blind randomized controlled study, 130 patients with BD were screened and 71 patients with BD were randomly allocated to the treatment (n=37) and control (n=34) groups. Finally, 32 and 30 patients in the treatment and control groups, respectively, completed the study. The study protocol was registered in the Iranian Registry of Clinical Trials (IRCT) with registration No. IRCT201511086975N5. Treatment and control groups received soft gels containing 1000 mg NS oil or 1000 mg placebo per day for 12 months, respectively. Disease activity using the Iranian Behcet’s disease dynamic activity measure (IBDDAM), total inflammatory activity index (TIAI) and Behcet’s disease current activity form (BDCAF) were evaluated in all patients before initiation of the trial and every 2 months, for 12 months.

**Results:**

Disease activity decreased in the study groups; difference between the two groups was not significant. No serious adverse events were seen in the treatment and control groups.

**Conclusion:**

NS oil at the dose of 1000 mg/day is not effective in controlling BD activity.

## Introduction

Behcet's disease (BD) is a multi-system chronic inflammatory disease characterized by recurrent oral aphthous ulceration (OAU), genital ulceration, uveitis and skin lesions. The highest prevalence of this disease was reported from Turkey (Mahr et al., 2008 ▶). Iran ranked second in terms of its prevalence (Davatchi et al., 2007 ▶). It is more common in the 2^nd^ to 4^th^ decades of life and men are more commonly affected compared to females (Khabbazi et al., 2014 ▶). The pathogenesis of BD is not fully understood; But, aberrant immune and inflammatory system activity triggered by environmental factors, perhaps use of microbial agents and vitamin D deficiency, in patients with a genetic susceptibility may be responsible (Alipour et al., 2017 ▶; de Smet et al., 2000 ▶; Dehghanzadeh et al., 2016 ▶; Esmaeili et al., 2011 ▶; Fortune et al., 1990 ▶; Khabbazi et al., 2014 ▶; Kolahi et al., 2015 ▶; Mazzoccoli et al., 2016 ▶; Suzuki et al., 2004 ▶; Yamamoto et al., 1993 ▶). 

*Nigella sativa* (NS) belongs to the Ranunculaceae Family. It has many nutritional and pharmacological properties including anti-inflammatory, anti-histaminic and anti-oxidant functions (Schleicher et al., 2000 ▶). NS is useful in the treatment of rheumatoid arthritis (RA) (Gheita et al., 2012 ▶). Its effectiveness is related to its ability in decreasing inflammatory cytokines such as interleukin 1β (IL-1β), IL-6, and tumor necrosis factor α (TNF-α), inhibition of cyclooxygenase and lipo- oxygenase, increasing the production of antioxidant enzymes and inhibition of nuclear factor kappa B (NF-KB) pathway (Budancamanak et al., 2006 ▶; Gheita et al. 2012 ▶; Sayed-Ahmed et al., 2012 ▶). On the other hand, NS decreases Th1/Th2 ratio and increases CD4/CD8 ratio. Moreover, NS increases IL-10 which has anti-inflammatory effects. In addition, it increases IL-3 production by leukocytes which reduces inflammation in RA and prevent bone and cartilage destruction though inhibition of TNF-α production and regulatory T cells expansion (Kheirouri et al. 2016 ▶). A randomized clinical trial on 42 patients with RA showed that NS 1000 mg/day reduces serum malondialdehyde (MDA) and nitric oxide (NO) and increases IL-10 compared with baseline (Hadi et al., 2016 ▶). Abdali (2009) ▶ compared topical NS with placebo in the treatment of oro-genital ulceration in 40 patients with BD. This study showed that topical NS decreases pain and healing time of oral and genital ulcers.

Due to the immunomodulatory and anti-inflammatory effects of NS, this herbal medicine may be effective in the treatment of BD. To the best of our knowledge, no studies have been conducted on the effect of systemic administration of NS in patients with BD. This randomized double-blind controlled study was designed to evaluate the effect of oral administration of NS oil on the treatment of BD.

## Materials and Methods

**Figure 1:**
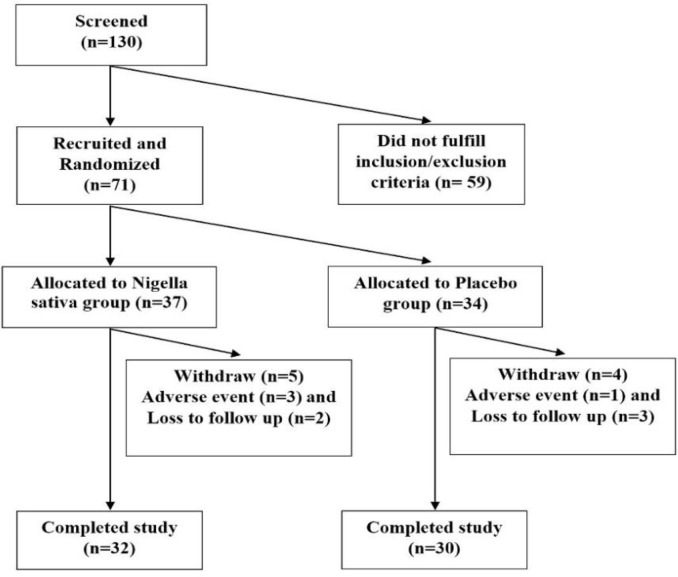
Recruitment and enrollment of the study participants

This randomized double-blind controlled study was done in the Connective Tissue Diseases Research Center (CTDRC) of Tabriz University of Medical Sciences (TUOMS) from December 2015 to May 2017. The study protocol was approved by the ethics committee of TUOMS and the trial registered in the Iranian Registry of Clinical Trials (IRCT) with the registration No. IRCT201511086975N5. BD was diagnosed based on International Criteria for BD (ICBD) (International Team for the Revision of the International Criteria for Behcet’s Disease, 2014). All patients had Azari ethnicity. In this trial, 130 patients with BD were screened and 71 patients were randomly allocated to the treatment (n=37) and control (n=34) groups (Figure. 1). Finally, 32 and 30 patients in the treatment and control groups, respectively, completed the study. Randomization was performed by RandList software 1.2 (http://www.randomisation.eu). Inclusion criteria were being 16 years old or older and having active BD. Exclusion criteria were consumption of NS oil supplements in the last 8 weeks, consumption of anti-oxidative preparations in the last 8 weeks, pregnancy, lactation, liver diseases and renal insufficiency. 

Treatment and control groups received one soft gelatin capsule containing 1000 mg NS oil or 1000 mg placebo per day for 12 months, 30 min before meal, respectively. Soft gelatin capsules containing NS oil and placebo were produced by Barij Essence Pharmaceutical Company, Iran. Disease activity in all patients was measured by the Iranian Behcet’s disease dynamic activity measure (IBDDAM) (Davatchi et al., 1991 ▶; Shahram et al., 2009 ▶), total inflammatory activity index (TIAI) (Davatchi et al., 1991 ▶; Shahram et al., 2009 ▶) and Behcet’s Disease current activity form (BDCAF) (Bhakta et al., 1999 ▶) before intervention and every 2 months for 12 months. If IBDDAM>2.5, TIAI>3.5 or BDCAF>1, then the disease was considered “active”. Patients and the assessing physician were blinded to the treatment regimen.

**Statistical analysis**

Statistical analysis was performed using SPSS version 16. Repeated measurements of analysis of variance (ANOVA), independent samples t-test, Chi-square or Fisher’s exact test were used for comparison between the studied groups. Data were expressed as mean±SD and frequency (%). P-values<0.05 were considered statistically significant.

## Results

Seventy-one patients with active BD were randomized to the treatment and control groups. Sixty-two patients completed the study (Figure 1). There was no significant difference in the demographic, clinical characteristics and medications of patients between treatment and control groups at the baseline (Table 1). After 12 months of treatment, disease activity as measured by IBDDAM, TIAI and BDCAF decreased in both groups with no significant differences between groups (Table 2). No serious adverse events happened in the treatment and control groups (Table 3). Three patients (two in the treatment and one in the control groups) discontinued medication because of severe dyspepsia. Cough happened in one patient from treatment group.

**Table 1:** Demographic and clinical characteristics and medications of patients at the baseline

	**Case ** **(N=37)**	**Control ** **(N=34)**	**P-value**
**Age (years)**	39.1±9.2	37.8±8.7	NS
**Male: Female**	22:15	18:16	NS
**Disease duration (years)**	11.2±6.1	10.48±5.4	NS
**Clinical manifestations**			
Oral aphthous ulcer (%) Genital ulcer (%) Skin lesions (%) Uveitis (%) Pathergy (%) Arthritis (%) Vascular involvement (%) CNS involvement (%) GI involvement (%)	36 (97.3) 21 (56.8) 14 (37.8) 16 (43.2) 12 (32.4) 7 (18.9) 5 (13.5) 2 (5.4) 0	33 (97.1) 17 (50.0) 16 (47.1) 16 (47.1) 13 (38.2) 6 (17.6) 5 (14.7) 3 (8.8) 1 (2.9)	NS NS NS NS NS NS NS NS NS
**Medications**			
Prednisolone (%) Colchicine (%) Azathioprine (%) Interferon ɑ (%) Methotrexate (%) Cyclosporine (%) NSAID (%) Mycophenolate Mofetil Remicade (%) Sulfasalazine (%) Cyclophosphamide (%)	27 (73.0) 13 (35.1) 10 (27.0) 6 (16.2) 5 (13.5) 1 (2.7) 1 (2.7) 1 (2.7) 2 (5.4) 2 (5.4) 1 (2.7)	28 (82.3) 10 (29.4) 12 (35.3) 2 (5.9) 7 (20.6) 1 (2.9) 4 (11.8) 2 (5.9) 3 (8.8) 0 (0.0) 0 (0.0)	NS NS NS NS NS NS NS NS NS NS NS

“The data of age was expressed as mean ± SD”, NS: Non-significant, CNS: Central nervous system involvement, NSAID: Nonsteroidal anti-inflammatory drug, GI: Gastrointestinal

**Table 2:** Treatment outcomes during 12 months

	**Case ** **(N=37)**	**Control ** **(N=34)**	**P-value**
IBDDAM at baseline	1.37±0.9	1.47±1.1	NS
BDCAF at baseline (	1.59±0.8	1.41±1	NS
TIAI at baseline	3.59±1.9	3.44±1.4	NS
IBDDAM at month 2	0.96±0.2	0.89±0.3	NS
BDCAF at month 2	0.88±0.2	1±0.2	NS
TIAI at month 2	2.75±0.9	2.87±1.1	NS
IBDDAM at month 4	0.64±0.2	0.77±0.3	NS
BDCAF at month 4	0.88±0.5	0.84±0.4	NS
TIAI at month 4	3.67±2.1	3.17±1.6	NS
IBDDAM at month 6	0.61±0.2	0.72±0.3	NS
BDCAF at month 6	0.91±0.9	0.75±1.1	NS
TIAI at month 6	2.23±1.4	1.98±1.2	NS
IBDDAM at month 8	0.56±0.4	0.62±0.5	NS
BDCAF at month 8	0.85±0.5	0.64±0.3	NS
TIAI at month 8	1.90±0.7	1.39±0.8	NS
IBDDAM at month 10	0.58±0.4	0.89±0.5	NS
BDCAF at month 10	0.78±0.4	0.67±0.5	NS
TIAI at month 10	1.81±0.7	1.41±0.8	NS
IBDDAM at month 12	0.61±0.9	0.66±1.1	NS
BDCAF at month 12	0.77±0.9	0.69±1.1	NS
TIAI at month 12	1.70±0.7	1.42±0.6	NS

“The data of IBDDAM, BDCAF and TIAI were expressed as mean ± SD"

IBDDAM: Iranian Behcet’s disease dynamic activity measure (IBDDAM); NS:Non-significant; SD: standard deviation; TIAI: Total inflammatory activity index; BDCAF: Behcet’s disease current activity form

**Table 3:** Side effects of treatment

	**Case (N=37)**	**Control (N=34)**
**Allergic reactions**	2	2
**Dyspepsia/Nausea/vomiting**	3	2
**Cough**	1	0
**Easy browsability**	1	1
**Others**	0	1

## Discussion

Altered innate immune function, cellular immunity and autoantibodies activity have been shown in the pathogenesis of BD. Autoreactive T cells play a critical role (Mazzoccoli et al., 2016). Th1 and Th17 cells and their related cytokines such as IL-2, IL-6, IL-8, IL-12, IL-17, IL-18, TNF-α, and interferon-γ (IFNγ) are dominant in BD (Mazzoccoli et al., 2016 ▶). Polymorphonuclear (PMN) leukocytes in patients with BD are activated and present increased motility and adhesion to endothelial cells (Doğan et al., 1994 ▶). Over-production of reactive oxygen species (ROS) including superoxide anions by activated neutrophils, decreased levels of superoxide scavenging activity in neutrophils and plasma, decreased activity of anti-oxidant enzymes and consequently impairment of oxidant/ anti-oxidant balance, higher levels of MDA in erythrocytes- which is an indicator of ROS-induced lipid peroxidation - have been shown in BD patients (Doğan et al., 1994 ▶; Köse et al., 1995 ▶). In addition, higher levels of prostaglandin-E2, Leukotriene C-4 (LTC-4), neutrophil NADPH oxidase and lower levels of total anti-oxidant status may play an important role in the pathogenesis of BD (Köse et al., 1995 ▶). 

Most of the therapeutic effects of NS are attributed to the presence of an alkaloid called thymoquinone (TQ) (Darakhshan et al., 2015). Anti-inflammatory and immunomodulatory effect of TQ includes: a) anti-oxidative effect due to the scavenging activity against superoxide anion, hydroxyl radical and singlet molecular oxygen; b) down-regulation of 5-lipoxygenase expression, and Leukotriene B4 (LTB4) and LTC4 production; c) decreasing levels of IL-1, IL-6, IL-8, TNF-α, and IFN-γ, and increasing level of IL-10; and d) suppression of NO over-production by macrophages) (Darakhshan et al., 2015).

Despite the initial expectation based on positive effect of NS to control BD activity and relief of clinical symptoms, our study showed no significant effect of 1000 mg/day NS on controlling BD activity. This result is contrary to a previous study reporting the effectiveness of NS on controlling RA symptoms (Gheita et al., 2012 ▶, Hadi et al., 2016 ▶) and treatment of its topical application on oro-genital ulcers of BD (Abdali, 2009 ▶). Gheita et al ▶. in a randomized controlled trial on 40 females with RA showed that administration of NS 1 g/day for 2 months, decreases disease activity (Gheita et al., 2012 ▶). Hadi et al. in another study, administered NS 1 g/day to RA patients and placebo to 25 patients for 2 months; authors reported that the disease activity was significantly reduced in the NS group (Hadi et al., 2016 ▶). To the best of our knowledge, this study was the first study to evaluate the effect of NS oil on activity index of BD. Ineffectiveness of NS oil on the BD activity index can be attributed to inappropriate dose of NS prescribed to patients. Thus, it is recommended to measure the effect of NS on inflammatory cytokines and immune mechanisms such as percentage of immune cell types and oxidative stress parameters in patients with BD using higher doses of NS. 

NS oil with dose of 1000 mg/d is not effective in control of BD activity.
